# An assessment of heart rate and blood pressure asymmetry in the diagnosis of vasovagal syncope in females

**DOI:** 10.3389/fphys.2022.1087837

**Published:** 2023-01-09

**Authors:** Rafał Pawłowski, Paweł Zalewski, Julia Newton, Agnieszka Piątkowska, Edward Koźluk, Grzegorz Opolski, Katarzyna Buszko

**Affiliations:** ^1^ Department of Biostatistics and Biomedical Systems Theory, Collegium Medicum, Nicolaus Copernicus University, Bydgoszcz, Poland; ^2^ Department of Exercise Physiology and Functional Anatomy, Ludwik Rydygier Collegium Medicum in Bydgoszcz Nicolaus Copernicus University in Torun, Bydgoszcz, Poland; ^3^ Department of Experimental and Clinical Physiology, Laboratory of Centre for Preclinical Research, Medical University of Warsaw, Warsaw, Poland; ^4^ Population Health Sciences Institute, The Medical School, Newcastle University, Newcastle, United Kingdom; ^5^ Department of Emergency Medicine, Wroclaw Medical University, Wroclaw, Poland; ^6^ 1st Chair and Department of Cardiology, Medical University of Warsaw, Warsaw, Poland

**Keywords:** vasovagal syncope, heart rate asymmetry, blood pressure asymmetry, Poincaré plot, heart rate variability (HRV)

## Abstract

**Introduction:** Heart Rate Asymmetry (HRA) describes a phenomenon of differences between accelerations and decelerations in human heart rate. Methods used for HRA assessment can be further implemented in the evaluation of asymmetry in blood pressure variations (Blood Pressure Asymmetry—BPA).

**Methods:** We have analyzed retrospectively the series of heartbeat intervals extracted from ECG and beat-to-beat blood pressure signals from 16 vasovagal patients (age: 32.1 ± 13.3; BMI: 21.6 ± 3.8; all female) and 19 healthy subjects (age: 34.6 ± 7.6; BMI: 22.1 ± 3.4; all female) who have undergone tilt test (70°). Asymmetry was evaluated with Poincaré plot-based methods for 5 min recordings from supine and tilt stages of the test. The analyzed biosignals were heart rate (RR), diastolic (dBP) and systolic Blood Pressure (sBP) and Pulse Pressure (PP). In the paper we explored the differences between healthy and vasovagal women.

**Results:** The changes of HRA indicators between supine and tilt were observed only in the control group (Porta Index *p* = 0.026 and Guzik Index *p* = 0.005). No significant differences in beat-to-beat variability (i.e. spread of points across the line of identity in Poincaré plot—SD1) of dBP was noted between supine and tilt in the vasovagal group (*p* = 0.433 in comparison to *p* = 0.014 in healthy females). Moreover, in vasovagal patients the PP was significantly different (supine: 41.47; tilt: 39.27 mmHg) comparing to healthy subjects (supine: 35.87; tilt: 33.50 mmHg) in supine (*p* = 0.019) and in tilt (*p* = 0.014).

**Discussion:** Analysis of HRA and BPA represents a promising method for the evaluation of cardiovascular response to orthostatic stressors, however currently it is difficult to determine a subject’s underlying health condition based only on these parameters.

## 1 Introduction

Understanding how the human heart rate varies over time and the methods to investigate this variability (heart rate variability—HRV) have been continuously developing over the past three decades. One of the methods of HRV assessment is the analysis of Poincaré plots (Woo et al., 1992; Brennan et al., 2001). The analysis of the plot shape allows measurement of the asymmetry in human heart rate in a variety of ways—it reveals that the human heart rate accelerates and decelerates in different ways. This phenomenon is also known as time irreversibility, since one can distinguish heart rate acceleration series (i.e., consecutive shortenings of time–distance between heartbeats) from deceleration series (prolongations of time–distance between heartbeats) by comparing them on the Poincaré plot. The asymmetric nature of HRV in healthy people was first recognized 15 years ago, although the exact cause of this phenomenon is not fully explained (De Maria et al., 2019). In particular, heart rate asymmetry (HRA) is interpreted as the observation that the amount of heartbeat accelerations prevails over that of decelerations in the majority of the healthy population (Porta et al., 2006). Furthermore, the contribution of decelerations and accelerations to HRV manifesting as an uneven distribution of points on the plot differs depending on the type of variability (i.e., the direction along the plot axes) (Guzik et al., 2006; Piskorski and Guzik, 2011).

The methods that have been implemented in HRV and HRA estimations may also be adopted in the analysis of other biosignals—such as blood pressure. The measurement of systolic blood pressure (sBP) and diastolic blood pressure (dBP) allows insights into blood pressure variability (BPV) and HRV. BPV is regulated by the autonomic nervous system (ANS) and provides information on the cardiovascular system control mechanisms (Electrophysiology Task Force of the European Society of Cardiology the North American Society of Pacing, 1996; Laitinen et al., 1999). The methods used in HRV and HRA assessments have been successfully implemented in the evaluation of BPV and its asymmetrical features (blood pressure asymmetry—BPA) (Guzik et al., 2010b). The results obtained by other researchers indicate the dependence on gender in the autonomic modulation of heart rate and in rhythmic BPV. Therefore, separate analyses for men and women are needed (Huikuri et al., 1996; Laitinen et al., 1999; Reulecke et al., 2016).

A comparative analysis of HRA in the groups suggests promising results in the diagnosis of some illnesses and health conditions, e.g., long QT syndrome or neonatal stress (Kramarić et al., 2019; Andrzejewska et al., 2022). It also gives deeper insight into the causes of other health conditions—since the heart rate is regulated by sympathetic and parasympathetic branches of ANS, the diseases related to the malfunctioning of these systems may affect the differences between the accelerations and decelerations of the heart rate (i.e., asymmetry). Some investigation have been performed over the past few years to evaluate the differences in HRA parameters in illnesses such as gastric cancer, diabetes, ADHD, and clinical depression and conditions such as stress, head-up tilt, or meditation (Porta et al., 2008; Guzik et al., 2010a; Tonhajzerova et al., 2012; Tonhajzerová et al., 2014; Parvaneh et al., 2015; Shi et al., 2019; Goshvarpour and Goshvarpour, 2021).

We focused our investigations on vasovagal syncope (VVS). VVS is defined as transient, reversible loss of consciousness which often results in a fall. An immediate cause of loss of consciousness is reversible global brain hypoperfusion of a short duration. VVS is one of the most common forms of the reflex syncope, i.e., those that are not caused by a serious illness of the cardiovascular or nervous system. The most common triggers of VVS include standing for a long period or standing up rapidly, heat exposure, sight of blood, fear, stress, or pain. Spontaneous syncope occurs in about half of healthy humans during their lives, and the neural pathways involved in the vasovagal response are probably present in all healthy people (Alboni et al., 2007). The VVS pathophysiology is not fully understood; however, some authors suggest that the ANS regulation disorder may be the main reason (Furlan et al., 2015). In some people, the orthostatic intolerance manifesting as a loss of consciousness is much more frequent than in others and significantly impacts their quality of life.

The head-up tilt test (HUTT) is the method of VVS diagnosis proposed by Kenny et al. (1986). It has been assumed that the syncope provoked during the test is the same as the syncope caused by a prolonged upright position in non-diagnostic situations. The test is performed on a special tilting table, and cardiac activity (ECG) and blood pressure are continuously monitored during the test. The HUTT is not classified as a gold standard in syncope diagnostics, and as such, there are numerous protocols of this examination. They differ in duration and the angle of tilt (between 45 and 90), type of back support, and the type of provocation. According to the current European Society of Cardiology guidelines, the recommended tilt angle is between 60° and 70°. The test enables the assessment of an individual’s susceptibility to VVS. However, 10%–15% of adults without a history of fainting will experience syncope during HUTT at 60–70°; likewise, there are people with orthostatic intolerance in whom tilt testing is unable to activate a typical vasovagal reaction (Brignole et al., 2000; Alboni et al., 2007). All test protocols consist of three steps: supine, tilt to a certain angle, and supine after tilt (Parry and Kenny, 1999). The protocol of the test can be modified by changing its duration and applying additional elements (e.g. controlled breathing, fist clenching, Valsalva maneuver or pharmacological provocation) (Parry and Kenny, 1999; Ducla-Soares et al., 2007).

Recent studies show promising results in diagnosing VVS using various HRA methods—the multistructure index (generalization of HRA indices) and the deceleration capacity (obtained from phase-rectified signal averaging) may serve as good discriminators of VVS (Makowiec et al., 2017; Zheng et al., 2020). We decided to further explore the physiology of VVS by analyzing HRV, BPV, and their asymmetries in order to better understand what is happening in people who are more sensitive to orthostatic stress. ANS functioning and blood pressure level may have a crucial role in the occurrence of VVS (Kochiadakis et al., 2004; Vaddadi et al., 2011). Therefore it is imperative to explore various parameters related to them in order to find differences between healthy people and those suffering from VVS. In that way, in the future, we could develop or modify the possible treatments and better understand the mechanism of VVS occurrence. In this study, we apply descriptors based on Poincaré plots to find their possible application in the diagnosis of vasovagal syncope in females. Moreover, we attempt to adapt those methods in the assessment of BPV and BPA in female patients who suffer from vasovagal syndrome.

## 2 Materials and methods

### 2.1 Signal acquisition and processing

**FIGURE 1:**
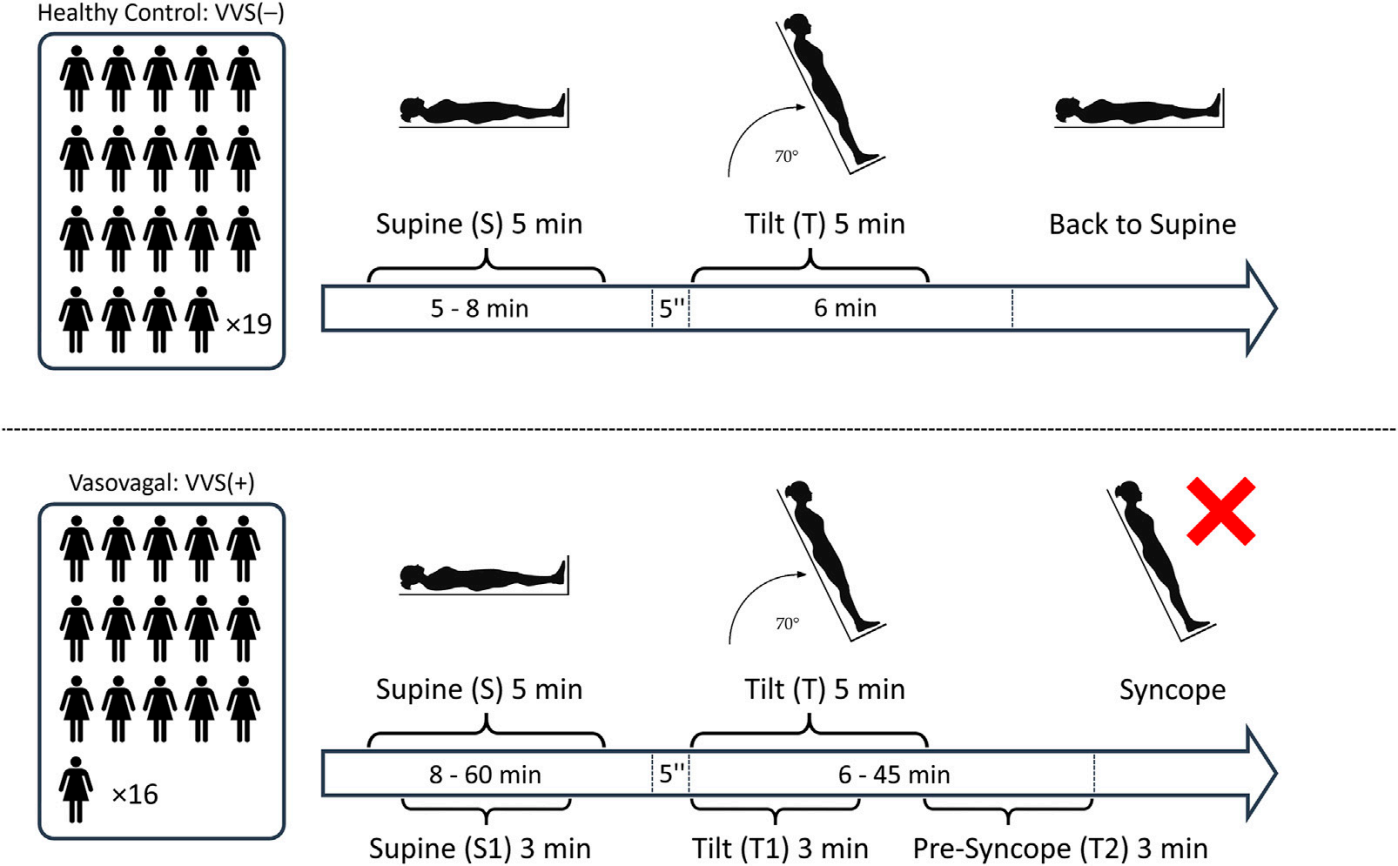
The schematic illustration of the study protocol: The number of participants in each study group and the segments of biosignals from the tilt test: S, 5 min supine; T, initial 5 min of tilt; S1, 3 min supine; T1, initial 3 min of tilt; and T2, pre-syncope phase (the last 3 min before the onset of syncope symptoms).

This retrospective study comprised two groups: 16 vasovagal female patients who developed syncope during the passive phase of the HUT test (VVS(+) group) and a control group of 19 healthy women (VVS(−) group) with no history of fainting and who had negative results in the upright HUTT phase for at least 6 min. The average age of patients in the VVS (+) group was 32.1 (±13.3), and average BMI was 21.6 (±3.8). The control group’s (VVS(−)) average age was 34.6 (±7.6), and average BMI was 22.1 (±3.4). There were no statistically significant differences between the groups with respect to age (*p* = 0.154) and BMI (*p* = 0.354). All participants underwent a 70 tilt test. The tests were performed with a Task Force Monitor (TFM) (CNSystem, Graz, Austria), which consists of two components: the first one is a moving table with a footboard and abdominal straps. The second one comprises devices for continuously monitoring the electrocardiogram (2-channels ECG, sampling rate 1 kHz), impedance cardiography (ICG), and blood pressure. The patients were fasting prior to the test. The day before the test, they also had to refrain from consumption of coffee and alcohol. The tests were performed in the morning in a dimly lit, quiet room, at a controlled temperature of 23–24°C. In case of vasovagal patients, the tilting procedure was as follows: after a rapid tilt to 70 (within 5 s), the patient remained upright for 45 min or until the syncope occurred. The ECG and blood-pressure recordings were then automatically converted by using TFM software into a series of the following beat-to-beat signals: heartbeat interval (RR), sBP, and dBP. The pulse pressure (PP) has been calculated as the difference between sBP and dBP. Five-min long recordings have been extracted from the signals as follows: from the middle part of the supine phase of the HUTT (S) and from the initial phase of the tilt phase (T)—right after the table reached the desired inclination. The detailed time window selection for the analysis is presented in Figure 1. The study protocol differed slightly between groups—the lengths of the S and T phases were different; however, the basic principles of the test (monitoring device, patient exclusion criteria, and test conditions) remained the same.

To ensure the selection of participants suitable for analysis, those cases where the number of possible non-sinus-origin heartbeats exceeded 5% of heartbeats in the recording were excluded. The detection was carried with the use of a quotient filter as in (Piskorski and Guzik 2005). After marking the incorrect RR interval, the values of blood pressure corresponding to it were also removed from the calculations. Furthermore, we excluded blood pressure recordings with more than 10% of missing values and where the recordings were too shorter than 5 min (and vasovagal patients who fainted in less than 6 min of being in an upright position). Thereby, 14 out of original 49 participants (study group and control) were excluded from the study. The total time of the remaining biosignal recordings in the supine was (after initial stabilization) 5–8 min in VVS(–) group and 8–60 min in VVS(+), and in the tilt phase, it was 6 min in VVS (–) and 6–45 min in the VVS (+) group.

### 2.2 Variability and asymmetry indices

**FIGURE 2:**
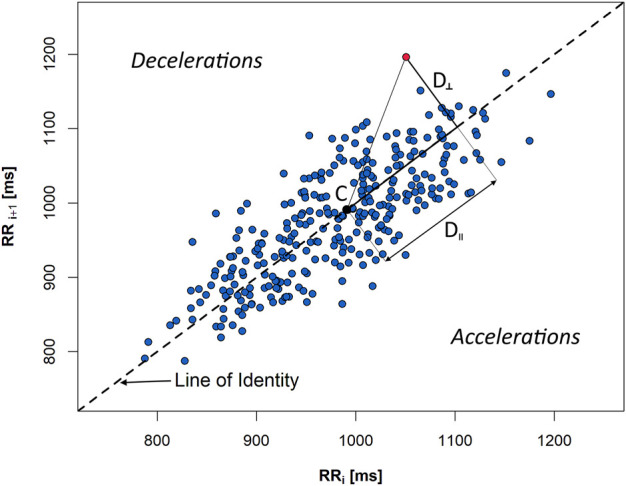
A Poincaré plot of a 5-min sequence of heartbeat intervals (RR) extracted from an ECG recording of a healthy person in supine. *D*_‖_ and *D*_⊥_ are, accordingly, in parallel and perpendicular distances, with regard to the line of identity, from the given plot point (red) to the centroid (C) of the figure created by all the points from the graph.

Numerous attempts have been made to assess HRV and HRA in the past which have included several non-linear descriptors such as Porta’s Index (PI), Guzik’s Index (GI), Area Index, Slope Index, and Ehlers Index (Ehlers et al., 1998; Guzik et al., 2006; Porta et al., 2006; Karmakar et al., 2015; Yan et al., 2017). Most of them are based on the Poincaré plot of the RR sequence (Figure 2).

The following variability and asymmetry indices have been employed in our study:

SD1 and SD2 describe the variability of the biosignal: beat-to-beat (also referred as short-term one)—SD1—and slow variability (also referred as long-term)—SD2 (Brennan et al., 2001). In order to avoid misinterpretation with long 24-h recordings, the terminology that we use in this paper is *beat-to-beat variability* for SD1 and *slow variability* for SD2 parameters. Thus, SD1 and SD2 measures describe HRV and BPV, depending on the kind of the signal they were calculated for.

SD1 is a spread of Poincaré plot points across the line of identity. It is calculated as a standard deviation of perpendicular distances (*D*_⊥_) of individual graph points to the line of identity (LI):

$$SD1=\sqrt{\frac{1}{n}\overset{n}{\underset{i=1}{\sum }}{D_{⊥}^{2}}_{i},}$$

where *n* is the total number of points which do not lie on the LI. SD2 is derived in a manner similar to SD1, whereas the distances (*D*_‖_) are measured along the LI to the centroid of the ellipse-like figure created by all the plot points. Therefore, SD2 is a spread of Poincaré plot points along the LI and signifies the slow variability (which can thus be calculated for biosignal recordings of any length) (Brennan et al., 2001).

We have used the following descriptors to assess the asymmetry:

*Guzik’s Index* (GI) is an index which allows the calculation of deceleration contribution into the SD1 (beat-to-beat HRV):

$$GI=\frac{{SD1d}^{2}}{{SD1}^{2}}\cdot 100,$$

where

$${SD1d}^{2}=\frac{1}{n}\overset{n_{+}}{\underset{i=1}{\sum }}{D_{⊥}^{2}}_{i}$$

is the mean of the squared perpendicular distances *D*_⊥_ (Figure 2) from the LI of the points, and *n*_*+*_ is the amount of points located above the identity line (decelerations) (Guzik et al., 2006).

*GI alternative for slow variability* (GI_S_) is the contribution of decelerations into slow HRV:

$${GI}_{S}=\frac{{SD2d}^{2}}{{SD2}^{2}}\cdot 100,$$

where SD2d is obtained analogous to SD1d with the difference that the distances *D*_‖_ are measured in parallel to the LI as projections onto LI—see Figure 2 (Piskorski and Guzik, 2011).

*Porta’s Index* (PI) is calculated as

$$PI=\frac{n_{-}}{n}\cdot 100,$$

where *n* stands for the number of points below the identity line (accelerations) (Porta et al., 2006). Thus, it may be understood as the percentage of accelerations in relation to all the heart rate variations (i.e., points that do not lie on LI).

In this study, the asymmetry is given by PI > 50, GI > 50, and GI_S_ < 50 (Piskorski and Guzik, 2011). Nowadays, the investigations are focused on creating new or redefining the already existing descriptors (e.g., redefined Guzik’s Index by Karmakar et al. (2012)) in order to assess HRA more precisely.

We used all the aforementioned parameters and adopted them afterward in BPV (by SD1 and SD2) and BPA (by GI, GI_S_, and PI) assessments. Therefore, in this study, the BPA approach refers to the asymmetry obtained analogous to that in Guzik et al. (2010b) rather than the inter-arm difference in blood pressure.

### 2.3 Phases of syncope development in vasovagal patients

The biosignal recordings from the VVS (+) group have been divided into three non-overlapping phases:

1) Rest in the supine position (3 min)—S1
2) Initial phase of the tilt (3 min)—T1
3) Pre-syncope phase (3 min)—T2

Afterward, we conducted the comparative analysis of the results obtained in each phase.

### 2.4 Statistical analysis

The normality of the result distribution has been verified with the Shapiro–Wilk test. The comparison of mean biosignal values and HRV and BPV parameters between the groups (at the same phase of HUTT) has been conducted using the Mann–Whitney *U* test. The Wilcoxon test has been employed to compare between HUTT phases within the same group. The asymmetry has been verified by GI and GI_S_, and the difference in proportions of the asymmetry occurrence has subsequently been examined with McNemar’s test. HRV and HRA indices have been compared with Friedman’s ANOVA followed by a pairwise *post-hoc* analysis (with Holm’s correction (Holm, 1979)). Afterward, the occurrences of slow and beat-to-beat asymmetry (determined by GI_S_ and GI, respectively) in biosignals have been verified in each phase of HUTT (namely S1, T1, and T2) and compared with the Cochran Q test with *post-hoc* analysis applied subsequently (with Holm’s correction).

The statistical analysis has been carried out with a significance level of *α* = 0.05 (significant results highlighted in red). All analyses were performed in R statistical software (Wickham, 2016; Piskorski, 2020; R Core Team, 2021). Each boxplot presents median, interquartile range, and marginal values within 1.5 times the interquartile range beyond quartiles reached by whiskers.

## 3 Results

### 3.1 Heart rate and blood pressure variability

The results of comparison of the mean RR interval, sBP, dBP, and *p*-values obtained for each subject between the study groups revealed significant differences in PP in both the supine (*p* = 0.037) and tilt (*p* = 0.029) phases of HUTT (Table 1). The dBP comparison yielded *p* = 0.076 in supine and *p* = 0.243 in tilt. The median of the average dBP in supine was greater in the VVS(−) group than in the VVS(+) group (76.1 and 68.8 mmHg, respectively). There was no significant difference in other biosignals between VVS(−) and VVS(+) groups in neither the supine nor the tilt phases of HUTT (RR and sBP comparison *p*-values >0.5).

**TABLE 1:** Descriptive statistics of the mean pulse pressure (PP) values recorded during the tilt test: 5 min in supine (S) and 5 min in tilt (T) in the vasovagal female patients’ group (VVS (+)) and control group (VVS (–)).

Mean PP (mmHg)	Min	Max	Q1	Median	Q3	Mean	SD
VVS (–)							
(S)	22.43	48.69	34.09	34.93	38.28	35.87	6.18
(T)	23.94	40.41	31.31	34.00	35.71	33.50	4.24
VVS (+)							
(S)	32.28	65.79	35.50	39.54	42.18	41.47	9.21
(T)	24.43	58.01	33.15	37.71	42.57	39.27	8.49

The in-between body position comparison (S vs. T) of the mean values within the same tested groups is presented in Table 2. We have observed significant changes in the heart rate and in the systolic and diastolic blood pressures during tilt in the vasovagal and control groups (all *p*-values <0.0001). The difference in pulse pressure was not significant (*p* = 0.096 in control and *p* = 0.231 in the vasovagal group).

**TABLE 2:** The comparison of the median of heart rate (RR interval) and blood pressure (systolic, sBP; diastolic, dBP; and pulse pressure, PP) variability descriptors between supine (S) and tilt (T) phases of the head-up tilt test in the vasovagal patients’ group (VVS (+)) and control group (VVS (–)). The variability descriptors are beat-to-beat and slow variability (SD1 and SD2, respectively) and mean values of the biosignal obtained for each subject.

	VVS(–)			VVS(+)		
	Median S)	Median T)	*p*-value	Median S)	Median T)	*p*-value
RR (ms)						
Mean	860.53	726.39	< 0.001	858.60	687.48	< 0.001
SD1	25.34	14.47	< 0.001	24.53	14.33	< 0.001
SD2	64.87	63.84	0.623	67.27	66.37	0.562
sBP (mmHg)						
Mean	114.55	122.62	< 0.001	110.51	123.93	< 0.001
SD1	1.17	1.62	0.096	1.33	1.89	0.528
SD2	5.88	8.54	< 0.001	7.21	10.90	0.065
dBP (mmHg)						
Mean	76.14	86.66	< 0.001	68.79	84.70	< 0.001
SD1	1.03	1.30	0.014	1.34	1.25	0.433
SD2	5.12	6.89	< 0.001	6.35	7.93	0.044
PP (mmHg)						
Mean	34.93	34.00	0.096	39.54	37.71	0.231
SD1	1.69	1.67	0.953	1.88	1.51	0.117
SD2	3.71	4.75	0.001	5.05	5.99	0.348

The analysis of SD1 and SD2 parameters in supine and tilt revealed significant difference in beat-to-beat variability (SD1) of diastolic blood pressure between supine and tilt only in the control group, which manifests as a greater dispersion of points across the line of identity on the Poincaré plot in tilt (*p* = 0.014; median: 1.03 in supine and 1.30 in tilt). We did not observe the same difference in SD1 of dBP between supine and tilt phases in the group of vasovagal patients (*p* = 0.433; median: 1.34 in supine and 1.25 in tilt). The difference in SD2 of dBP has been observed in both the VVS(–) and VVS(+) groups (*p* < 0.001 and *p* = 0.044, respectively), which may be noticed as Poincaré plot elongation: from 5.12 to 6.89 mmHg in the control group and from 6.35 to 7.93 mmHg in the vasovagal group.

**FIGURE 3:**
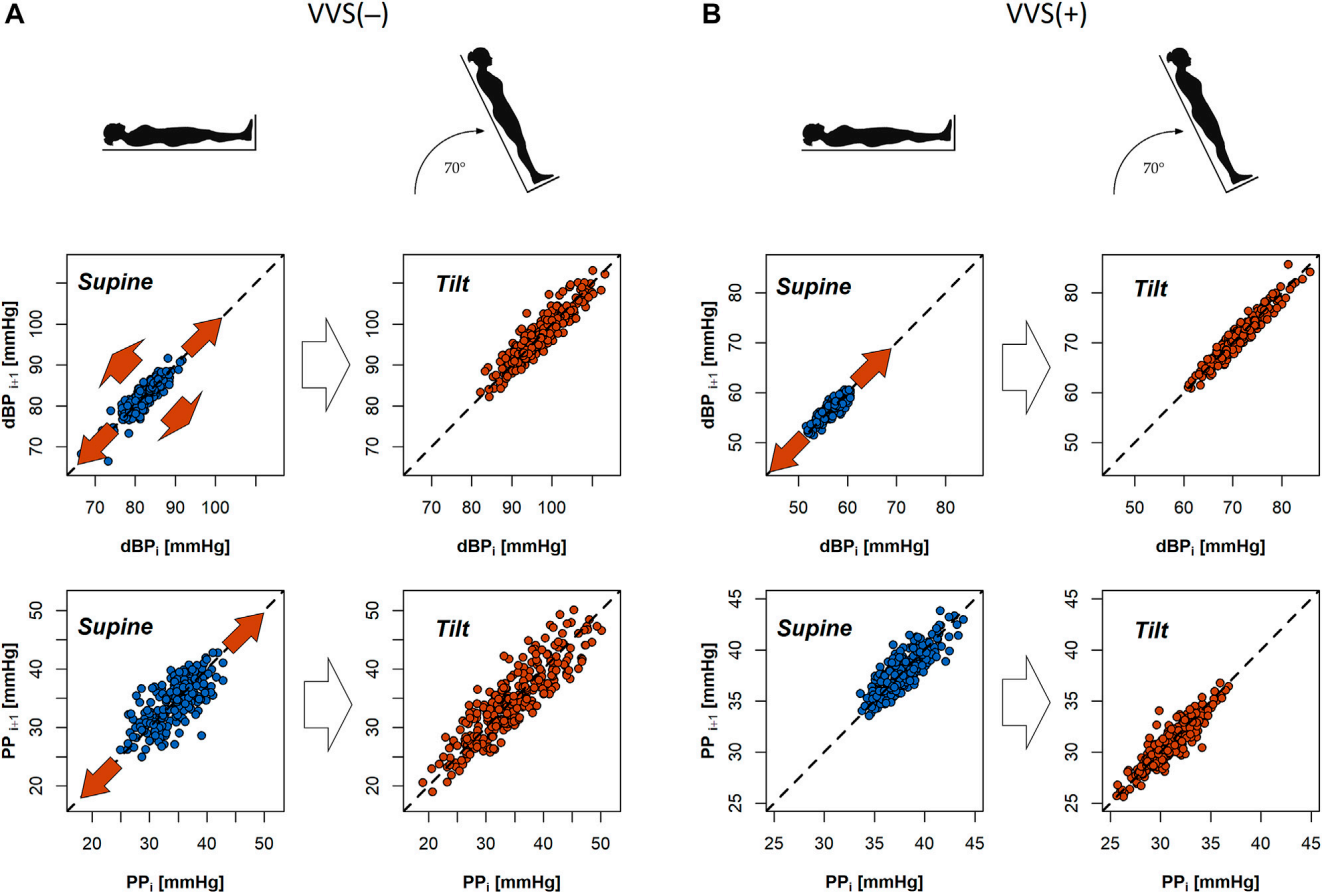
Poincaré plots of diastolic blood pressure (dBP) and pulse pressure (PP) biosignals of a subject from the control group **(A)** and a vasovagal patient **(B)** during the tilt test. Red arrows in the graphs indicate that the direction of dispersion of points across and alongside the line of identity in the plot (SD1 and SD2 descriptors) changes between the supine and tilt phases.

Cardiovascular system response to tilt in the VVS(–) group yielded a significant difference in slow PP variability (SD2 parameter; *p* = 0.001). The same transition was not observed in the VVS (+) group (*p* = 0.348). Those differences in patient reactions to HUTT are visible on exemplary Poincaré plots of dBP and PP signals from the studied subjects (Figure 3).

The analysis of the systolic BPV results showed significant difference in SD2 only in the VVS (–) group: *p* < 0.001 (*p* = 0.065 in vasovagal group). The SD1 differences were not significant: VVS (–): *p* = 0.096 and VVS (+): *p* = 0.528.

### 3.2 Heart rate and blood pressure asymmetry

We have observed HRA (GI > 50) occurrence in 31.6% of the VVS (–) group in supine and 63.2% in tilt and in 56.3% and 75.0% in supine and tilt of the VVS (+) patients, respectively. GI asymmetry in sBP has been observed in 36.8% (S) and 57.9% (T) in the control group, whereas in the vasovagal group, the BPA has been registered in 31.3% (S) and 56.3% (T) of patients. We did not observe significant differences in proportions of asymmetry occurrence between supine and tilt in neither the GI nor GI_S_ in any of the measured biosignals (all *p*-values > 0.1). dBP asymmetry (GI > 50) has been observed in 18.8% (S) and 50.0% (T) of vasovagal patients (*p* = 0.13 in 5-min recordings).

The comparison of GI, PI, and GI_S_ asymmetry parameters between VVS (+) and VVS (–) (within the same phase of HUTT) showed that the differences in BPA indices were not significant (all *p*-values > 0.1 in sBP, dBP, and PP). The comparisons of HRA indices yielded values with *p* = 0.056 for GI in supine and *p* > 0.1 in other cases.

**FIGURE 4:**
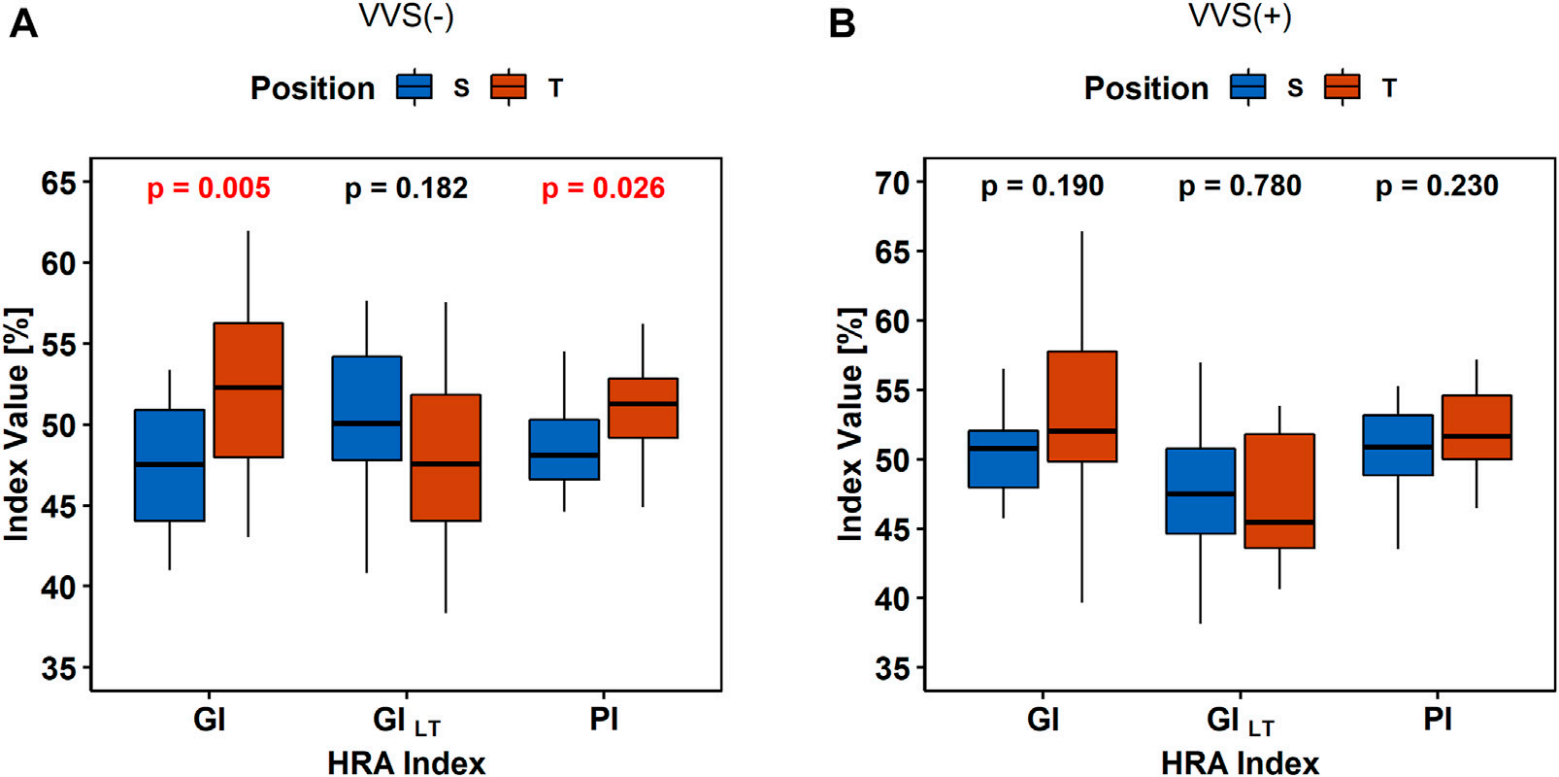
Comparison of heart rate asymmetry indices (Guzik’s Index, GI; Porta’s Index, PI; and slow-term Guzik’s Index, GI_S_) between the supine (S) and tilt (T) phases of the tilt test in the control group **(A)** and in vasovagal patients **(B)**.

The results of the same descriptor (GI, PI, and GI) value comparisons in the heart rate between S and *P* are presented in Figure 4.

**FIGURE 5:**
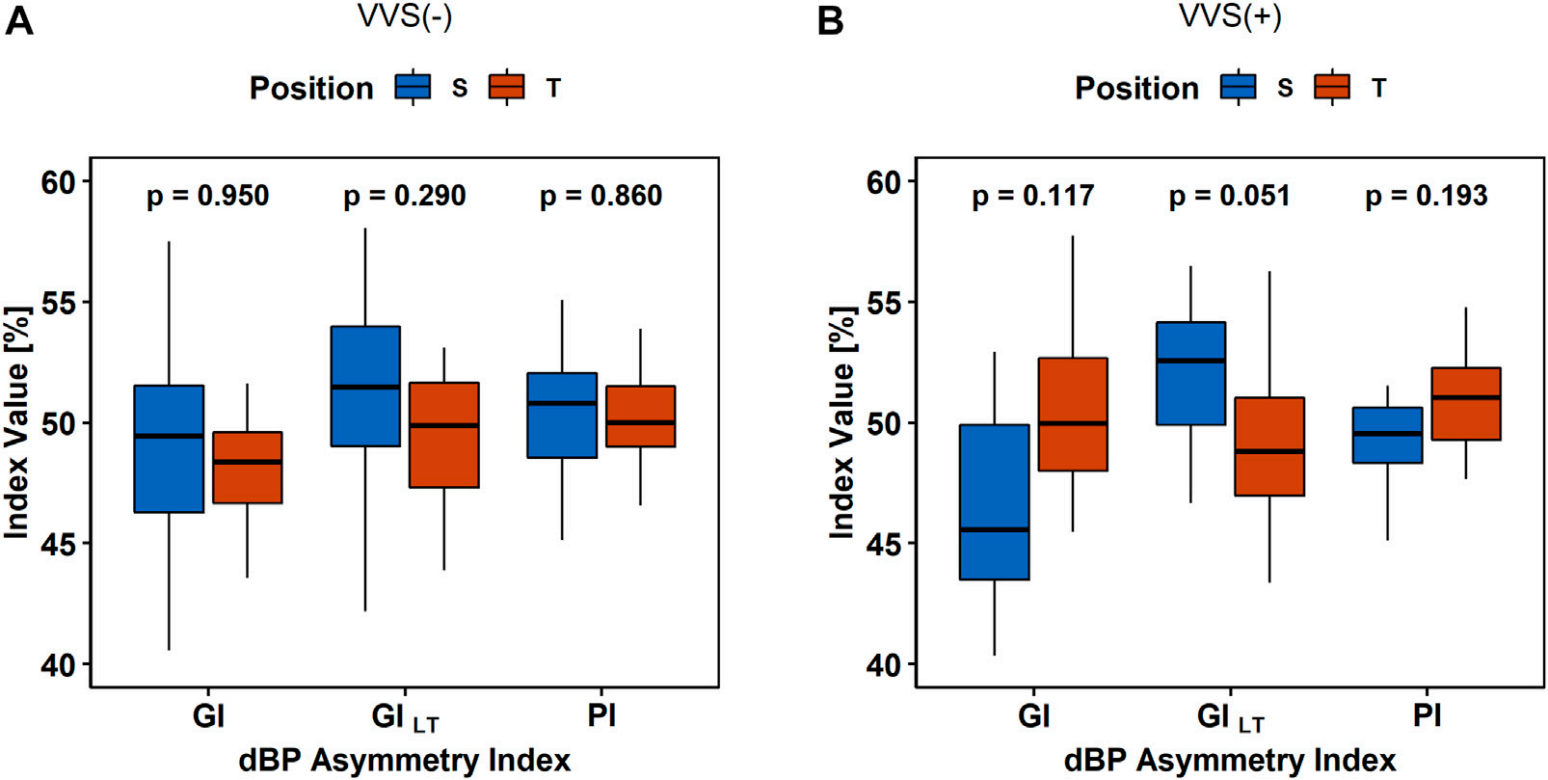
The comparison of asymmetry indices (Guzik’s Index, GI; Porta’s Index, PI; and slow-term Guzik’s Index, GI_S_) of diastolic blood pressure (dBP) in **(A)** the control group (VVS (–)) and **(B)** vasovagal females (VVS (+)) between the supine (S) and tilt (T) phases of the head-up tilt test.

The BPA analysis between HUTT phases did not show any differences in the sBP and PP asymmetry indices (all *p*-values > 0.1) between S and T in the VVS (–) and VVS (+) groups. The diastolic BPA results are presented in Figure 5.

### 3.3 Syncope development by variability and asymmetry measures

The comparison of variability in heart rate and blood pressure and their asymmetry indices between the supine (S1), initial part of tilt (T1), and pre-syncope phase (T2) carried in the VVS (+) group has been presented in Table 3.

**TABLE 3:** Changes in variability and asymmetry of heartbeat interval (RR) and systolic, diastolic, and pulse blood pressure (sBP, dBP, and PP, respectively) recorded in the vasovagal female group. Comparison between the supine (S), initial part of tilt (T1), and pre-syncope phase (T2) of HUTT conducted on biosignal variability measures—beat-to-beat and slow variability (SD1 and SD2) and its asymmetry indices: Guzik’s Index (GI), slow-term Guzik’s Index (GI_S_), and Porta’s Index (PI).

Descriptor	Friedman’s *p*	*Post-hoc p* ^a^		
		S1–T1	S1–T2	T1–T2
RR				
Mean	< 0.001	0.001	0.001	0.001
SD1	< 0.001	0.001	0.001	0.001
SD2	0.001	0.938	0.064	0.023
PI	0.7788	1.000	1.000	1.000
GI	0.3679	0.734	0.970	0.970
GI_S_	0.6456	1.000	1.000	1.000
sBP				
Mean	< 0.001	0.002	0.485	0.003
SD1	0.087	0.970	0.970	0.422
SD2	0.047	0.109	0.149	0.149
PI	0.087	0.698	0.096	0.096
GI	0.829	1.000	1.000	1.000
GI_S_	0.444	0.776	0.133	0.109
dBP				
Mean	< 0.001	0.001	0.083	0.002
SD1	0.105	0.816	0.533	0.084
SD2	0.185	0.149	0.073	0.856
PI	0.829	0.375	0.375	0.177
GI	0.047	0.512	0.512	0.856
GI_S_	0.087	0.979	0.049	0.027
PP				
Mean PP	0.099	0.157	0.157	0.660
SD1	0.087	0.313	0.177	0.177
SD2	0.002	0.149	0.623	0.017
PI	0.269	0.673	0.673	0.673
GI	0.443	0.940	0.940	0.940
GI_S_	0.779	0.734	1.000	0.734

^a^ Holm’s correction.

**FIGURE 6:**
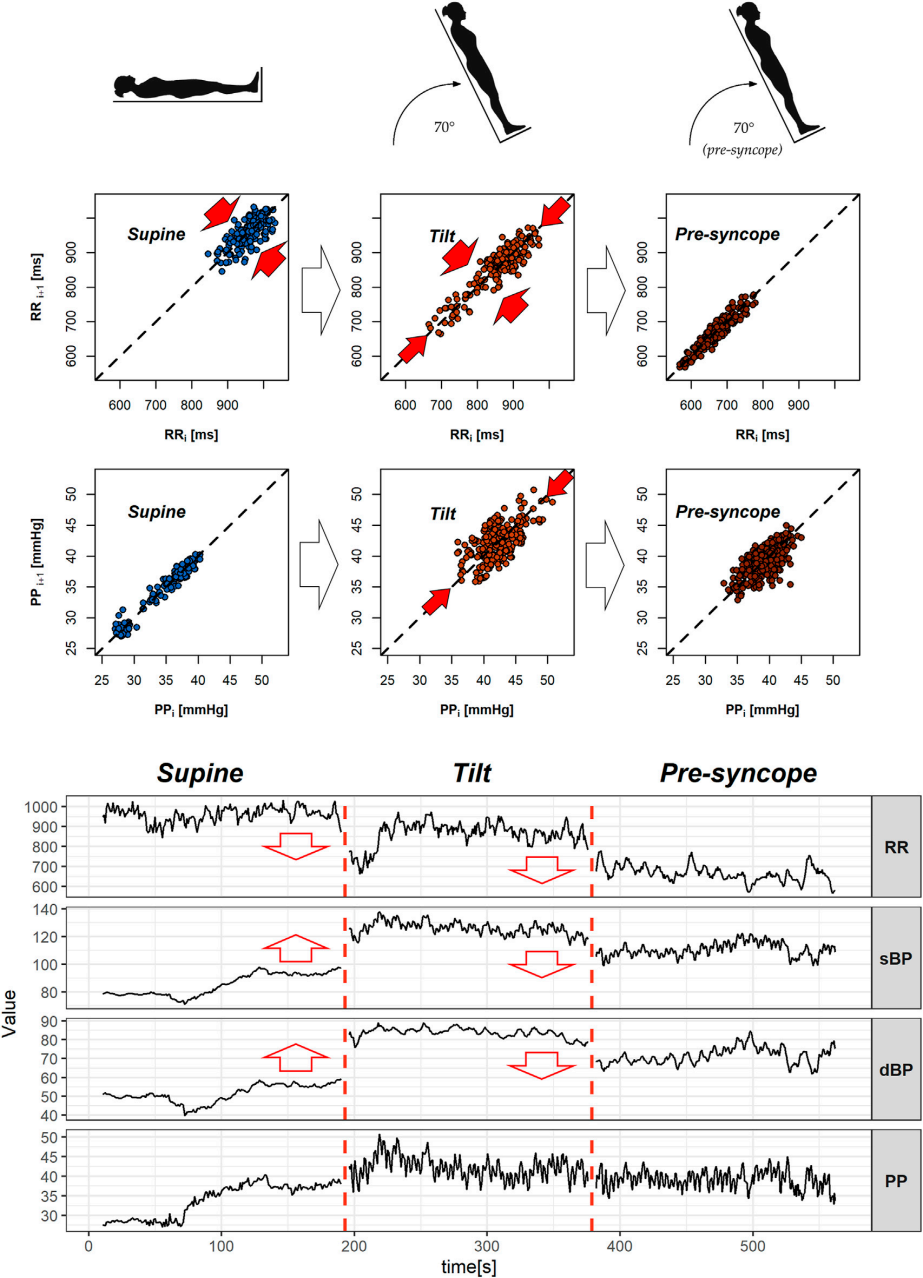
Poincaré plots with exemplary heartbeat intervals (RR), pulse pressure (PP) series, signal recordings of heart rate (RR), and blood pressure (sBP, dBP, and PP) of vasovagal female patients. The figure presents 3-min segments of the head-up tilt test phases—from the left: supine (S1), tilt (T1), and pre-syncope in tilt (T2). Red arrows in the Poincaré plots indicate significant difference in variability measures (beat-to-beat: SD1 and slow: SD2), while ones in signal graphs represent changes in the mean value between the featured and the following phases.

The changes in variability parameters occurred only in RR and PP signals, while changes of asymmetry indices were not confirmed in the *post-hoc* analysis in any of the biosignals. Exemplary biosignal recordings and Poincaré plots with observed variability differences in S1, T1, and T2 stages of the test are presented in Figure 6.

The mean RR distance value lowers gradually during the successive phases of the test: from 867.00 m in supine to 707.95 m in tilt (*p* = 0.001) and reaches 597.48 m in the pre-syncope phase (*p* = 0.001). The means of sBP and dBP are different during the T1 phase in comparison to S1 (*p* = 0.002 in sBP and *p* = 0.001 in dBP) and T2 (*p* = 0.003 in sBP and *p* = 0.002 in dBP) phases of the tilt test. The measures of position of the mean values of the signal are presented in Table 4.

**TABLE 4:** The measures of the positions (median and mean) of mean values of heartbeat intervals (RR) and blood pressure (systolic, sBP; diastolic, dBP; and pulse pressure, PP) biosignals measured in vasovagal patients during 3 min of supine (S1), tilt (T1), and pre-syncope (T2) phases of the head-up tilt test.

Median (mean)	S1	T1	T2
RR (ms)	867.00 (844.94)	707.95 (695.64)	597.48 (597.12)
sBP (mmHg)	111.13 (109.10)	125.91 (125.03)	109.64 (111.26)
dBP (mmHg)	69.77 (67.49)	87.33 (86.62)	72.98 (73.76)
PP (mmHg)	39.48 (41.61)	37.78 (38.42)	35.14 (37.5)

The comparative analysis of asymmetric occurrence in heart rate and blood pressure is presented in Table 5. We observed a significant difference only in the diastolic BPA occurrence between supine and the initial 3 min of the tilt phase of HUTT (*p* = 0.024). The details of dBP asymmetry occurrence in the VVS (+) group during the test are presented in contingency tables (Table 6) in order to illustrate the differences, especially between S1 and T1 groups.

**TABLE 5:** Asymmetry proportion comparisons in the analyzed biosignals: the asymmetry occurrence between supine (S1), the initial part of tilt (T1), and the pre-syncope phase of tilt (T2) in the head-up tilt test in vasovagal patients obtained by Porta’s Index (PI > 50) and Guzik’s Index (GI > 50 and GI_S_ < 50).

Measured biosignal	Asymmetry indicator	*p*-values	*Post-hoc p* ^a^		
			S1–T1	S1–T2	T1–T2
RR	PI > 50	1.000	1.000	1.000	1.000
sBP		0.121	0.514	0.176	0.514
dBP		0.105	0.514	0.102	0.514
PP		0.339	0.472	0.705	0.635
RR	GI > 50	0.779	1.000	1.000	1.000
sBP		0.558	0.951	1.000	1.000
dBP		0.037	0.024	0.068	0.763
PP		0.607	1.000	1.000	1.000
RR	GI_S_ < 50	0.895	1.000	1.000	1.000
sBP		0.121	0.480	0.076	0.359
dBP		0.174	0.705	0.411	0.176
PP		0.282	0.395	0.635	0.635

^a^ Holm’s correction.

**TABLE 6:** Contingency tables of GI asymmetry occurrence in diastolic blood pressure in the vasovagal patients’ group. Diastolic blood pressure asymmetry occurrence is marked as 1, absence as 0. Head-up tilt test phases: S1, supine; T1, tilt (initial 3 min); and T2, pre-syncope 3 min.

S1–T1			S1–T2			T1–T2		
	T1 (1)	T1 (0)		T2 (1)	T2 (0)		T2 (1)	T2 (0)
S1 (1)	2	0	S1 (1)	1	1	T1 (1)	3	6
S1 (0)	7	7	S1 (0)	7	7	T1 (0)	5	2

## 4 Discussion

In this study, we have focused on the difference in response to orthostatic stress in vasovagal syncope rather than a direct distinction between cohorts; however, the significantly different PP in both the supine (*p* = 0.037) and tilt (*p* = 0.029) phases of HUTT between the study group and control is noteworthy. However, normal pulse pressure oscillates around 40 mmHg, which indicates that the difference might be a result of relatively low pulse pressure in the control group.

Recent research indicates cardioneuroablation (denervating the heart) as an effective tool for VVS treatment (Vandenberk et al., 2022). The efficiency of this method suggests that one of the main factors responsible for VVS is an abnormally increased vagal tone. The study of deceleration capacity (which is an HRA index related to the vagal activity) strengthens this conclusion (Zheng et al., 2020; Tu et al., 2022). In the case of cardioinhibitory VVS, the patients after cardioneuroablation treatment had lower mean RR and significantly changed HRV parameters toward parasympathetic withdrawal (Piotrowski et al., 2022). However, other research indicates the usefulness of the heart rate Poincaré plot shape analyses in VVS diagnosis (Yuan et al., 2022).

Although the detailed explanation of the mechanism of the asymmetry in the heart rhythm is unknown, it is considered that HRA is related to the balance between sympathetic and parasympathetic activations in ANS. Klintworth et al. (2012) pointed out that the different response times of both systems may be one of the causes of HRA. The HRA research shows that some of its markers are not correlated with the respiratory rhythm; however, different results in asymmetry are obtained depending on the breathing pattern (Klintworth et al., 2012; Wang et al., 2013; De Maria et al., 2021). The accumulation of blood in the lower body caused by gravitational force during the first 60 s of tilt leads to an initial drop in blood pressure, which, through the baroreceptor reaction, triggers the ANS: the initial phase of heart rate acceleration depends on vagal withdrawal, while the subsequent one is linked to sympathetic activation (Shannon et al., 1986). A further increase in blood pressure, temporarily even above the baseline values, stimulates the baroreceptor reflexes, leading to a slowdown in the heart rate. The baroreflex loop (the effect of the RR length on SBP and *vice versa*) may be disturbed in VVS patients; however, to demonstrate this, a comparative analysis would have to be conducted for HRA and baroreflex effects. The baroreflex response is influenced by the sign of the blood pressure variations—baroreflex compensates more efficiently where sBP rises instead of dropping. The positive association between this baroreflex asymmetry and HRA markers (GI and PI) during active standing in healthy people has been observed and proposed as one of the determinants of asymmetry in HRV (De Maria et al., 2019).

### 4.1 Heart rate and blood pressure variability

The observed property of HRV response to tilt in healthy women manifests as a change only between supine and tilt in beat-to-beat HRV, unlike in our previous study which was conducted on a larger group of healthy men where the changes of both beat-to-beat and slow HRV have been noted (Pawłowski et al., 2021). The increase of SD2 in HRV during the tilt phase only in males may be a signal of weaker adaptation to the upright position by the female cardiovascular system and explains why women are more affected by VVS than males (Alboni et al., 2021). However, despite the similar age range in both (male and female) groups, the group size difference makes it hard to draw conclusions about differences in HRV by gender from this reasoning.

The absence of dBP beat-to-beat variability increase during tilt in the VVS (+) group may be the manifestation of a weaker adjustment to an upright position, which is one of the components contributing to fainting. The observed increase of PP’s long-term variability only in healthy volunteers suggests that the problem of vasovagal reaction to the orthostatic stress also happens in PP modulation.

We do not draw conclusions from results of the SD1 and SD2 variabilities in sBP. The comparison of those parameters between supine and tilt draws attention to differences between the VVS (–) and VVS (+) groups by SD2 and equalizes them by SD1 (Table 2); however, the difference in *p*-value comparison between supine and tilt of SD1 in the studied units (*p* = 0.096 and *p* = 0.528) is much more substantial than in SD2 (*p* < 0.001 and *p* = 0.065). The results of the SD1 comparison were not statistically significant in any group, while the comparison of SD2 yielded *p* < 0.05 in one group. There is a possibility that the analysis conducted on larger study groups will bring differences in sBP variability between the S and T phases of HUTT analogous to dBP.

### 4.2 Heart rate and blood pressure asymmetry

The research carried out so far on HRA and BPA allows for only statistical assessment of those features in healthy population. The study conducted on 227 healthy young volunteers (19–31 years old; 97 females) by Guzik et al. (2010b) shows the occurrence of HRA in 62%–83% of participants, depending on the estimation method, and 75%–82% of individuals (71–81% females) in systolic BPA, depending on the method. According to these results, the absence of asymmetry in heart period or beat-to-beat blood pressure variation does not necessarily indicate health problems. The occurrence of HRA is less common in older people—probably because of lower cardiovascular efficiency due to age and lower sensitivity of baroreceptors, and thus, reactivity of the sympathetic and parasympathetic nervous systems in the elderly (Mitchell Gary F. et al., 2004; De Maria et al., 2019). The relatively small heart rate GI obtained in the present study in the VVS (–) group (mean ± SD: 47.3 ± 4.0) may be a reason for the non-significant difference of this index between the groups in supine (*p* = 0.056). The results of HRA and BPA occurrences in the control group gave us better insight into the nature of this phenomenon’s appearance from a statistical point of view. Piskorski and Guzik (2012) reported the occurrence of HRA (GI > 50) in healthy people (at rest in the supine position) at a level of ∼80% in numerous studies (Piskorski and Guzik, 2007; Piskorski and Guzik, 2011; Piskorski et al., 2019). In our study, the tendency is the opposite: HRA is present in 31.6% of healthy subjects. This result shows that the occurrence of HRA considered as a health indicator should be developed and applied to a larger group. The time spent by the subject in asymmetry is another factor that could have had an impact on this—it is possible to extract non-HRA heartbeat subsequence from longer ones which manifest asymmetrical tendencies (Piskorski et al., 2019; Pawłowski et al., 2021). The same consideration applies in the case of low occurrence of BPA in our study (36.8% in healthy females in supine) despite the independence of both phenomena (HRA and BPA) revealed by Guzik et al. (2010b).

The aforementioned independence of both phenomena should be more closely examined in the future. HRA and BPA may be contradictory to each other—the same feature of heart rate decelerations is observed during blood pressure increase (i.e., GI > 50). There is a possible negative correlation of HRA and BPA since the study was carried out on 28 healthy young subjects (21 female) by Chladekova et al. (2012) and reported an increase of HRA GI and decrease of systolic BPA GI after tilt; however, the study protocol included active standing instead of passive tilt. The transfer of the BPV asymmetry signal from the heart rate to blood pressure *via* baroreflex response presumed in the same study seems quite probable.

The simultaneous presence and absence of GI and PI increasing after changing the body position in the studied groups may be caused by the correlation between GI and PI, as observed by Klintworth et al. (2012). Numerous studies report an increase of this HRA parameter (GI) after the body position changes to the upright position (Porta et al., 2008; Tonhajzerová et al., 2014; De Maria et al., 2019). Thus, HRA GI, as an indicator of deceleration contributions to the beat-to-beat variability of the heart rate, may rate the vagal withdrawal rather than sympathetic activation in the tilt phase. Such an interpretation might explain slower increase of HRA GI in the VVS (+) group after tilt (Figure 4B) as a sign of insufficient vagal tone reduction, given that the vasovagal response follows stimulation of the vagus nerve and suppression of the sympathetic response.

### 4.3 Syncope development by variability and asymmetry measures

The multistructure index (a generalization of asymmetry indices) derived by Makowiec et al. (2017) gives promising perspectives of HRA and BPA estimations as a diagnostic tool in vasovagal syndrome; however, our study takes gender difference into account and tests different asymmetry measures. Furthermore, the initial seconds after the positional change are included in the analysis of the current study. Morillo et al. (1997) claimed that VVS patients have normal initial responses to upright tilt, and vasovagal physiology begins before apparent pre-syncope. Our study reaffirms those observations as we did not note any differences in mean values of the biosignal during the tilt phase except PP (which likewise differed in supine). Moreover, we did not observe any difference in the proportion of diastolic BPA between supine and tilt in the 5-min-long recordings of VVS patients; however, a comparison of the 3-min recording between the supine and initial stages of the tilt phase yielded significant difference (Table 5 and Table 6). This contradictory result suggests that the initial seconds of tilt might also be crucial in the analysis of body reaction to orthostasis and points to the importance of how asymmetry is defined—the difference between studied groups in response to tilt is not visible in the analysis of raw GI values. We have noted significant differences between initial and pre-syncope stages of HUTT in variability of heart rate and PP (Table 3 and Figure 6) in vasovagal females. Olufsen et al. (2006) proposed a model allowing the prediction of the dynamics of heart rate regulation during postural change and reported that baroreflex modulation does not return to the steady state during the first minute after standing up in hypertensive elderly people. Their results show that the greatest differences between healthy young and elderly people and also elderly people with hypertension are revealed during the first minute after standing upright (reduced baroreflex sensitivity with aging and even further reduced baroreflex function for hypertensive subjects). Bloomfield et al. (1997) compared two methods of posture changing (actively standing up and head-up tilting) and showed that the differences visible in the initial stage of orthostasis disappear when analyzing the averaged 5-min fragments. Further research over the initial seconds right after a postural change in people suffering from VVS is advised due to the possible occurrence of signs which indicate problems with the ANS regulation at that moment.

## 5 Conclusion

The weaker increase of HRA markers during the tilt phase in vasovagal females may indicate an insufficient vagal withdrawal in ANS response to orthostasis. In vasovagal women, the increase of the SD1 parameter (the measure of differences between successive blood pressure records variations) in the tilt is absent. The extension of a measure of monotonic heart rate series length (SD2) during tilt may be determined by gender. Increased pulse pressure in females may be an indicator of vasovagal syndrome. The ANS reaction in the initial 60 s of the upright position should be considered during the VVS diagnosis. The dBP variations in VVS patients as a body answer to the orthostatic stressor are more apparent than sBP variations. It is difficult to determine a subject’s health condition based only on GI and PI heart rate asymmetry markers.

## 6 Limitations

The relatively small study groups make it hard to draw solid conclusions from the current results, and, therefore, we suggest treating this paper as an indication of the possibilities and limitations of the tested methods. Also, for this reason, we did not divide the subjects into age groups. In the analysis, we assumed that the signals maintain their stationarity in the short segments and we did not analyze this issue separately. Past studies indicated that HRV (and thus, HRA) may be modulated by the menstrual cycle, which was not taken into account in the present work (Vallejo et al., 2005; Bai et al., 2009; Tenan et al., 2014).

## Data Availability

The raw data supporting the conclusions of this article will be made available by the authors, without undue reservation.
